# Resilienz Kritischer Infrastruktur im Krankenhaus: Kategorisierung und Quantifizierung als Grundlage der Optimierung

**DOI:** 10.1007/s00101-023-01318-9

**Published:** 2023-08-16

**Authors:** Rico U. Hübner, Cornelia Küsel, Jörg W. Oestmann

**Affiliations:** 1 Klinik für Radiologie, Charité – Universitätsmedizin Berlin, Charitéplatz 1, 10117 Berlin, Deutschland; 2 Abteilung E, Sanitätsakademie der Bundeswehr, Neuherbergstr. 11, 80937 München, Deutschland; 3 Fakultät für Informatik, Universität der Bundeswehr München, Werner-Heisenberg-Weg 39, 85579 Neubiberg, Deutschland

**Keywords:** Systemanalyse, Krisenmanagement, Kritische Infrastruktur, Risikoanalyse, Katastrophenvorsorge, System analysis, Crisis management, Critical infrastructure, Risk analysis, Disaster preparedness

## Abstract

**Hintergrund:**

Die Kritische Infrastruktur in Krankenhäusern (KRITIS) ist durch die Auswirkungen der COVID-19-Pandemie und auch der Ereignisse in der Ukraine in den Fokus der Resilienzforschung gerückt. Die vorliegende Grundlagenuntersuchung analysiert Gesamtzusammenhänge, kategorisiert und quantifiziert diese. Bisherige Forschungen untersuchten Schadenslagen begrenzten Ausmaßes mit geringer KRITIS-Beteiligung: Worst-Case-Studien fehlen.

**Fragestellung:**

Ist es möglich, ein kategorisiertes und gewichtetes Modell zur Selbstbewertung der Resilienz Kritischer Infrastruktur in Krankenhäusern für das exemplarische Szenario eines längeren überregionalen Stromausfalls zu erstellen und zu bewerten?

**Material und Methoden:**

Das Forschungsdesign ist explorativ. Mit Expert*innen aus 8 Kliniken unterschiedlicher Versorgungsstufen wurde in einer qualitativen Systemanalyse das Modell anonym erstellt, gewichtet und getestet. Der Resilienzindex wurde dann mithilfe von adaptierten Interdependenzanalysen berechnet

**Ergebnisse:**

Es wurden 7 Kategorien und 24 Unterkategorien identifiziert. Die Netzersatzanlage (E1) hat die größten Auswirkungen auf alle anderen Bereiche. Das Pflegepersonal (P2) ist für seine Arbeit am stärksten von allen anderen abhängig. Die kritischsten Elemente sind das Lagezentrum/*der Führungsstab (Z1) und Technisches Personal (P3), von denen das gesamte System abhängt. Aus den gewichteten Einzelelementen lässt sich eine Gesamtresilienz für ein Krankenhaus berechnen (Resilienzindex).*

**Diskussion:**

Die Kategorisierung und Quantifizierung der KRITIS in Krankenhäusern mit dem Ziel der Resilienzmessung und Optimierung ist möglich. Das erarbeitete Modell erlaubt eine schnelle Anpassung an sich wandelnde Ausgangslagen und kurz- sowie mittelfristig realisierbare Resilienzsteigerungen.

## Abstract (en)

**Background:**

Critical infrastructure (CRITIS) in hospitals has become the focus of resilience research due to the impact of the COVID-19 pandemic and also the events in Ukraine. This foundational research examines overall contexts, categorizing and quantifying them. Previous research examined limited scale damage situations with little CRITIS involvement: Worst case studies are missing. The vulnerabilities of the CRITIS of one or more countries will likewise be a prime target for attack in current and future conflicts or criminal extortion, this is especially true in the healthcare sector. Therefore, detailed research with a black swan scenario is necessary in this field.

**Objective:**

The aim of the study was to create and validate a categorized and weighted model for the self-assessment of the resilience of critical infrastructure in German hospitals at different levels of care before the exemplary scenario of a prolonged supraregional power blackout.

**Material and methods:**

Using an explorative design, experts from 8 hospitals of different care levels performed an expert-based qualitative system analysis to develop, weight and test the model. The resilience index was then calculated using adapted interdependence analyses in a Vester influence matrix.

**Results:**

A total of 7 categories and 24 subcategories of hospital CRITIS were identified. There are several key elements: rank 1 of active elements is the emergency power system (E1), and for passive elements, it is the nursing staff (P2). This means that the emergency power system has the greatest impact on all other areas and the nursing staff are most dependent on all others for their work. The most critical elements, because they are most intertwined in the overall system, are the situation center/command staff (Z1) and technical staff (P3), on which the entire system depends. From the weighted individual elements of CRITIS, an overall resilience for a hospital can be calculated (resilience index). The developed model can be used by hospital crisis experts as part of a self-assessment to provide a basis for risk management, financial planning, technical planning, personnel planning or crisis and disaster management.

**Conclusion:**

The categorization and quantification of critical infrastructure (CRITIS) in hospitals with the aim of resilience documentation and optimization is possible. The model that has been developed allows rapid adaptation to changing initial situations and increases in resilience that can be realized in the short and medium term.

Emergency and crisis preparedness is a dynamic process, which has been combined here with the further development of critical infrastructure. Consequently, there can be no final state to be achieved but only a certain best possible framework within which the hospital as a business enterprise can operate. The classification of the categories in the model must also be constantly further developed and adapted to the current status. Once the explorative and qualitative basic research has been completed, it is necessary in a further step to subject the model, which has been validated by experts, to a broader review. Ideally, this will be done using quantitative methods and a significantly larger sample.

Die Bewertung der Widerstandsfähigkeit Kritischer Infrastruktur (KRITIS) innerhalb von Krankenhäusern gegenüber äußeren Ereignissen ist bis dato nur für wenig technisierte Entwicklungsländer im Fokus der Forschung. Aufgrund aktueller weltweiter politischer wie medizinischer Herausforderungen ist hier ein Umdenken zu mehr Krisenvorsorge und Anpassungsfähigkeit notwendig. Die vorliegende Studie legt den Grundstein zu einer Analyse der KRITIS hochvernetzter komplexer Kliniksysteme in Industrieländern.

## Hintergrund und Fragestellung

Die COVID-19-Pandemie hat vielen Bereichen der Kritischen Infrastruktur (KRITIS) weltweit aufgezeigt, dass ein bedeutsamer Ausfall von Personal nicht nur die direkte Bereitstellung von Versorgungsleistungen beeinträchtigt, sondern auch wichtige Lieferketten nachhaltig stört. Die Kritischen Infrastrukturen werden ebenso bei Konflikten oder kriminellen Erpressungen ein bevorzugtes Angriffsziel darstellen – das gilt insbesondere für den Gesundheitssektor [2]. Der Begriff Resilienz (lat. „resiliere“) bezeichnet für Krankenhäuser deren Fähigkeit, auch unter extremen Rahmenbedingungen den Regelbetrieb aufrechtzuerhalten. Kritische Infrastrukturen betreffen „Organisationen oder Einrichtungen mit wichtiger Bedeutung für das staatliche Gemeinwesen, bei deren Ausfall oder Beeinträchtigung nachhaltig wirkende Versorgungsengpässe, erhebliche Störungen der öffentlichen Sicherheit oder andere dramatische Folgen eintreten würden“ [3]. Krankenhäusern kommt im Bereich KRITIS eine Doppelrolle zu, indem sie einerseits Bestandteil der nationalen KRITIS sind, gleichzeitig *aus* (Elementen von) KRITIS bestehen und *mit* KRITIS interagieren müssen. Die Vorgaben länderspezifischer Regelungen reflektieren Art und Umfang der Bedrohung nicht in ausreichendem Maße [18]. Im Dezember 2022 veröffentlichte die Bundesregierung mit den Eckpunkten für das KRITIS-Dachgesetz einen Ausblick auf kommende Verpflichtungen für Krankenhausbetreiber zum Risiko‑, Krisen und Resilienzmanagement [4]. Hier setzt die durchgeführte Untersuchung an.

### Risiken und Bedrohungen für KRITIS

Zahlreiche Publikationen befassen sich mit den Ursachen des großflächigen Ausfalls von KRITIS, was jedoch für die Planung der Versorgungssicherheit im Krankenhaus eher nachrangig ist. Szenarien, deren Schwere und Verlauf nicht vorhersagbar sind (sog. Black Swan), können nur durch eine flexible Reaktionsmöglichkeit sowie die Kenntnisse und Fähigkeiten der beteiligten Akteure beherrscht werden [15]. Eine Studie des Büro für Technikfolgen-Abschätzung beim Deutschen Bundestag von 2011 zeigt, dass das Gesundheitssystem in Deutschland bei einem großflächigen Stromausfall bereits nach 24 h ein kritisches Niveau erreicht hätte und nach einer Woche vor seinem Zusammenbruch stünde, da neben Elektrizität auch Lebensmittel, Betriebsstoffe, Wasser und Kommunikationsmittel nicht nachversorgt werden könnten [11]. Aufgrund der geringen Eintrittswahrscheinlichkeit wird der überregionale Totalausfall Kritischer Infrastruktur in der Krankenhausalarm- und -einsatzplanung (KAEP) in der Regel nicht berücksichtigt [1]. Folglich ist kaum jemand auf das Szenario eines hybriden Angriffs [6] auf Krankenhäuser vorbereitet, bei dem sich ein Aggressor mit Planungsaufwand und zeitlichem Vorlauf die Schwachstellen des gesamten Systems sucht und zum für den Angegriffenen ungünstigsten Zeitpunkt zuschlägt. Die bewährten Methoden des Risikomanagements, die ein genaues Ereignis und dessen Eintrittswahrscheinlichkeit erfordern, sind hier nur sehr begrenzt anwendbar. Die Lösung kann daher nur in einer flexiblen Systemresilienz liegen, die ereignisunabhängig ist. Der Risikoanalytiker Nassim Nicholas Taleb nennt diesen Ansatz zur Bewältigung von Black-Swan-Ereignissen Antifragilität [19]. Um diese theoretischen Überlegungen für die praktische Anwendung im Krankenhaus nutzbar zu machen, sind weitere Forschungen und wissenschaftlich fundierte Präventionsansätze notwendig [12, 13].

Basierend auf der Studie „Kritische Infrastrukturen – Resilienz als Mindestversorgungskonzept“ (KIRMin) [7] wurde 2020 ein Leitfaden „Analyse von Interdependenzen zwischen KRITIS“ [5] veröffentlicht, der kommunalen Anwendern das Konzept der Interdependenzanalyse praxisnah und kontextbezogen erörtert. Einen ähnlichen Forschungsansatz verfolgt die vorliegende Arbeit:

Ziel ist die Erfassung der komplexen Faktoren Kritischer Infrastruktur eines Krankenhauses und der Wechselbeziehungen wichtiger Schlüsselelemente vor dem Hintergrund eines hybriden Szenarios. Daher wurden KRITIS-Faktoren im Krankenhaus identifiziert, kategorisiert, gewichtet und daraus abgeleitet ein Index vorgeschlagen, mithilfe dessen es möglich ist, Resilienz zu bewerten, vergleichen und zu optimieren.

## Studiendesign und Untersuchungsmethoden

Für die Erstellung eines Modells der KRITIS-Faktoren, welche mit individuellen Gewichtungen hinterlegt ist, wurden im Detail folgende Schritte durchgeführt:

1. expertengestützte qualitative Systemanalyse (systematische und spezifische Literaturrecherche, Befragung von Fachleuten, Ortsbesichtigung),
2. Entwurf des Systemmodells mit verschiedenen Fachexpert*innen,
3. Validierung I zur Komplexitätsreduktion mit Fachexpert*innen aus Krankenhäusern,
4. Erstellung des Systemmodells,
5. Validierung II und Interdependenzanalyse mit Fachexpert*innen aus Krankenhäusern (wie 3.),
6. Auswertung, finales Modell, Berechnung von Indizes,
7. Ermittlung einer Formel zur Abbildung der Gewichtungen,
8. Erstellung eines Berechnungsbeispiels für den Resilienzindex.

Da die systematische Literaturrecherche wie auch die geführten Informationsgespräche mit Fachleuten aus Bundesamt für Bevölkerungsschutz und Katastrophenhilfe (BBK), Technischem Hilfswerk (THW), Bundeswehr, Katastrophenschutzbehörden und Krankenhäusern offenlegten, dass zu dem Problembereich wenige Vorarbeiten vorhanden sind, wurde die Entscheidung für ein exploratives Forschungsdesign getroffen.

Dies beinhaltet die Analysen von Einzelfällen und mehrstufige Vergleiche der Ergebnisse untereinander (sog. Induktiver Ansatz, Delphi-Verfahren). Die zu untersuchenden Elemente definieren sich z. T. erst im Forschungsprozess selbst [8].

Die Modellbildung für die Berechnung des Resilienzindex basiert auf einer Abbildung der (beobachteten) Realität in einem Systemmodell, dessen Abstraktion, Erklärung und Vereinfachung mit Bezug auf den beabsichtigten Zweck, um final ein aussagekräftiges technisch-soziales Modell zu erhalten [9].

Für das gewählte Beispielszenario eines Stromausfalls im Winter wurde in einer expertengestützten qualitativen Systemanalyse [21] erarbeitet, welche Elemente Kritischer Infrastruktur innerhalb eines Krankenhauses eine Rolle spielen, um dessen Funktionsfähigkeit als Gesamtsystem zu erhalten.

Um eine repräsentative Auswahl an Fachexpert*innen für die beiden Validierungsschritte I und II zu gewährleisten, wurde aus dem Krankenhausverzeichnis des Statistischen Bundesamtes eine Zufallsstichprobe gezogen, die nach Trägerschaft und Versorgungsstufe unterschied. Da hier insgesamt 12 verschiedene^1^ Kombinationen möglich sind, wurde dies auch als Umfang der Untersuchung festgelegt. Die Expert*innen sollten im Bereich der direkten Verantwortung für Faktoren von KRITIS im Krankenhaus über mehrjährige nachweisbare Erfahrung verfügen, da die Repräsentativität der quantitativen Einzelfallanalyse hierdurch nachzuweisen ist [10].

Bei der Validierung I erfolgten die Einteilung kritischer Faktoren in Kategorien und Unterkategorien und die grafische Darstellung als Modellentwurf. Dieser wurde bei der Validierung II mit den Expert*innen besprochen und verfeinert. Vor den Interviews wurde ein Pretest durchgeführt, bei welchem der Interviewleitfaden auf seine Verständlichkeit hin geprüft wurde. Mit den Expert*innen wurde aus Datenschutzgründen ein anonymisiertes Simultanprotokoll^2^ geführt und in der Folge aus den Protokollen die finale Reihung der Unterkategorien ermittelt.

Die erhaltenen Unterkategorien wurden in einer Einflussmatrix nach Vester [20] erfasst und bewertet. Dieses Analysetool für vernetzte Systeme bildet Einfluss und Beeinflussung der Einzelkomponenten sowohl numerisch als auch grafisch ab und erlaubt hierbei die Identifikation aktiver, puffernder, reaktiver und kritischer Elemente wie auch die Darstellung von Wechselwirkungen. In der Folge lassen sich für jedes Element dessen Bedeutung im Gesamtsystem und eine individuelle Gewichtung ableiten.

Aus der befüllten Einflussmatrix ergaben sich für jede der Unterkategorien Punktsummen in der Aktivspalte (wie sehr werden andere Elemente der KRITIS beeinflusst) und in der Passivspalte (wie sehr wird die Unterkategorie selbst von anderen Elementen der KRITIS beeinflusst).

Für eine weitere Interpretation der Daten wurden Interaktionsindex und Aktivitätsindex für jede Unterkategorie bestimmt [14]. Der Interaktionsindex (II) errechnet sich aus Aktivsumme (AS) mal Passivsumme (PS) und ist ein direkter Indikator für den Grad der Vernetzung eines Elements. Je höher der II, umso größer die Vernetzung. Der Aktivitätsindex (AI) wird berechnet aus dem Quotienten AS ÷ PS. Ein AI größer als 1 verweist auf ein aktives Element, welches andere beeinflusst und ein AI kleiner als 1 auf ein von anderen beeinflusstes reaktives Element. Je größer die Abweichung, desto eindeutiger die Wirkung.

Die Gewichtung (G) der einzelnen Unterkategorien wurde ermittelt durch Multiplikation der Abweichungsstärke des AI von seinem ausgeglichenen Wert mit dem Betrag des Interaktionsindex +1. Dies beruht auf Vesters Annahme, dass neben den kritischen auch deutlich aktive und deutlich reaktive Elemente eine spürbare Wirkung auf das Gesamtsystem haben [20]. Die abschließende Addition von 1 verhindert, dass durch Multiplikation mit 0 eine Verzerrung von Elementen stattfindet, die einen ausgewogenen AI aufweisen.

$$G^{UK}=\left(AS^{UK}\times PS^{UK}\right)\times \left| 1-\left(AS^{UK}\div PS^{UK}\right)\right| +1$$

Gleichzeitig wurden die Aussagen der Expert*innen aus den Interviews mit den rechnerisch ermittelten Werten der Matrix verglichen, um evtl. Inkonsistenzen zu identifizieren.

Die Visualisierung erfolgte in einem Punktdiagramm nach Vester [20], welches die Faktoren nach ihren Eigenschaften sortiert in 4 Quadranten als aktiv, puffernd, kritisch und reaktiv abbildet.

## Ergebnisse

Eine systematische Literaturrecherche zum Stand der Forschung mit Suchzeitraum 2011–2021 in den Datenbanken base-search.net und EBSCO^3^ erbrachte keine verwertbaren Ergebnisse^4^. Suchbegriffe waren [resilience AND hospital or clinic AND war, or crisis or disaster]. Gesucht wurden die Begriffe in Titel und Abstract der Publikationen. Eingeschlossen wurden Publikationen, die in Deutsch oder Englisch publiziert wurden und sich mit infrastrukturellen oder organisatorischen Maßnahmen zum Schutz kritischer Infrastruktur in Krankenhäusern oder vergleichbaren Einrichtungen (z. B. Reha-Kliniken) auseinandersetzen. Der Hospital Safety Index (HSI) wurde zwecks Abgrenzung in die Modellbildung einbezogen, scheidet aber als Vergleichsmaßstab aus, da er für Entwicklungsländer konzipiert wurde.

### Modellentwurf.

Die aus Literatur und den Informationsgesprächen gesammelten Erkenntnisse zu KRITIS-Kategorien wurden in technischen Sinneinheiten visualisiert. Mit den Expert*innen wurden die theoretischen Inhalte ergänzt und hierbei auch externe Fachleute (THW, Bundeswehr, Hochschulen, BBK, DRK) einbezogen. Nach Auswertung und Sortierung aller Quellen konnten für die KRITIS in Krankenhäusern 6 Kategorien und 49 Unterkategorien gebildet werden. Während der Komplexitätsreduktion wurden durch die Expert*innen artverwandte oder komplementäre Unterkategorien zusammengefasst oder gestrichen.

### Validierung.

Die Experteninterviews zur Validierung des Modells wurden vom 06.10.2021 bis zum 05.11.2021 durchgeführt. Insgesamt beteiligten sich von den ausgewählten 12 Interviewpartnern 8 Kliniken, sowohl vonseiten der Geschäftsführung als auch mit Haustechnik- und Krisenmanagementexperten.

### Validierung I.

**Figure 1:**
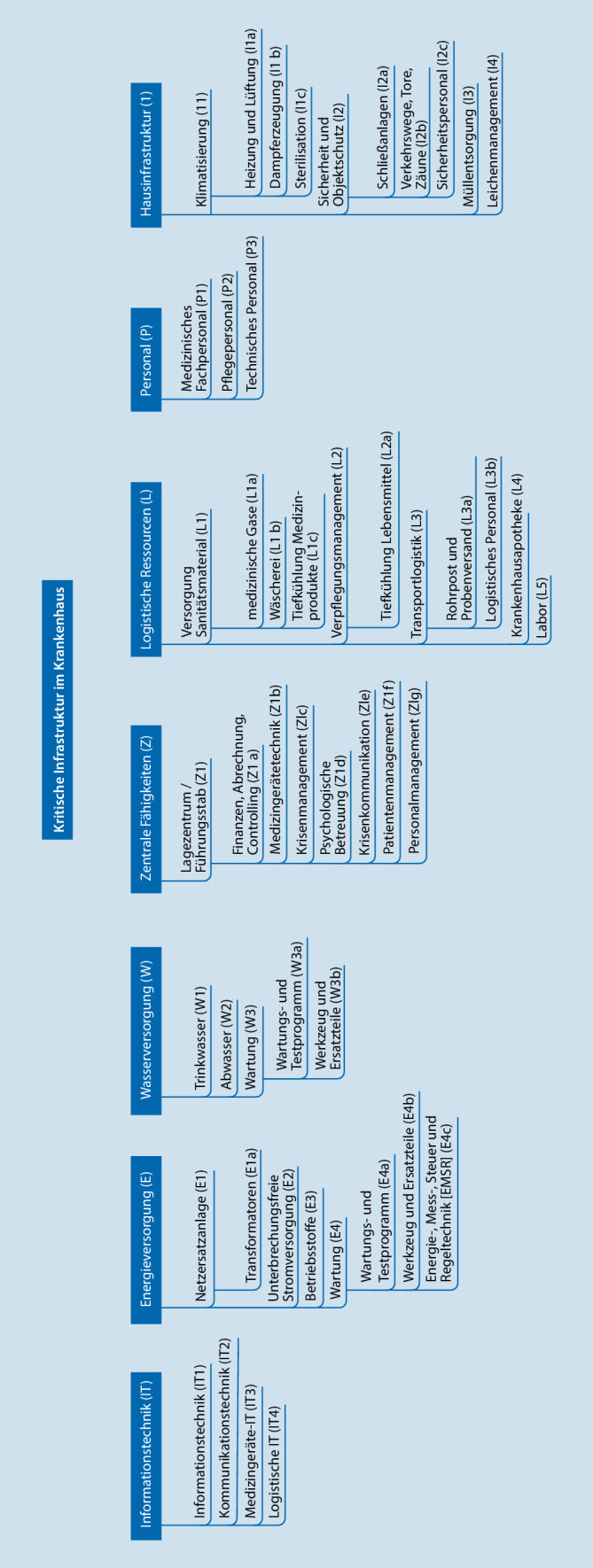

Der Modellentwurf wurde durch Konsensfindung zwischen den Expert*innen umgeordnet, und die Unterkategorien wurden von 49 auf 24 reduziert (Abb. 1).

### Validierung II.

Das Modell wurde final abgestimmt und den Expert*innen Fragen zur Erfahrung mit Notfallplanung bei KRITIS in Krankenhäusern gestellt. Im weiteren Verlauf wurde durch alle beteiligten Kliniken eine Einflussmatrix befüllt. Sechs der 8 beteiligten Kliniken blieben bis zum Ende der Studie dabei und füllten die Matrix vollständig aus.

### Erfahrungen der KRITIS-Expert*innen

Allen befragten Expert*innen sind ein oder mehrere einschlägige *Dokumente* zu KRITIS in Krankenhäusern bekannt, die sie jedoch als nicht ausreichend bewerten, wenn es um die Vorbereitung lang anhaltender Krisenszenare geht. Ein *Vergleich der Vorsorgebestrebungen* mit anderen Krankenhäusern und ein Austausch mit Technik-Expert*innen werden meist innerhalb einer Konzerngruppe oder regional innerhalb einer Stadt oder eines Landkreises realisiert, leider oft nicht behördlich organisiert, sondern eigeninitiativ.

Die *Mindestbedarfe an Ressourcen* im Krisenfall werden von allen befragten Krankenhäusern erhoben. Im Regelfall findet hier eine jährliche Evaluierung statt, auch im Zusammenhang mit stattfindenden Umwelt-Audits. Die Notstromversorgung wird im Schnitt ein- 2‑mal jährlich getestet, wobei ein Krankenhaus der Maximalversorgung mit monatlichen Testläufen des kompletten Systems an der Spitze liegt. In der Spanne liegt die logistische Reserve der Krankenhäuser bei 24–72 h; im Mittel können 36 h ohne externe Nachversorgung abgedeckt werden.

Die Expert*innen sehen Angriffe auf die IT und Stromausfälle als *schlimmste denkbare Szenarien* für die KRITIS eines Krankenhauses.

*Notfallübungen* mit Bezug zur KRITIS werden in jedem der befragten Krankenhäuser durchgeführt. Häufigkeit und Umfang der Übungen unterscheiden sich. Im Mittel liegen die Übungen ein bis 3 Jahre auseinander. Meist werden Hilfsorganisationen der Städte oder Landkreise eingebunden, auch um Kommunikationswege festzulegen und zu erproben. Hierbei traten auch gefährliche Defizite durch unterschiedliche technische Ausstattungen der Organisationen zutage, die dazu führten, dass eine Verständigung nicht möglich war.

Als wichtigste äußere *Einflussfaktoren auf die Resilienz* wurden die Finanzierung der Krankenhäuser sowie lückenhaften Gesetze und Vorschriften zu Bevorratung, Reserven und Vorsorge benannt.

Die *ideale Notstromversorgung* wird als ein redundantes System aus mehreren verteilten Diesel-Netzersatzanlagen beschrieben. Die Versorgung mit Betriebsstoffen sollte durch die Kommune in enger Kooperation mit dem örtlichen Energieversorger sichergestellt sein, da man dort ebenfalls Bedarf an Betriebsstoffen haben wird.

*Kritische Einzelelemente*, die Kettenreaktionen auslösen können und daher besondere Berücksichtigung in der Notfallplanung erfahren sollten, sind elektrische Bauteile als Sonderanfertigungen wie Netzteile und Transformatoren. Im Falle eines Ausfalles könnte die Neubeschaffung hier mehrere Wochen in Anspruch nehmen.

In der Kategorie Personal wird ein Seuchenausbruch als existenziell bewertet, da sich für besonders qualifiziertes Fachpersonal kurzfristig kein Ersatz finden lässt.

IT und Wasserversorgung wurden als vulnerabelste, Energieversorgung als robusteste, Personalmanagement als komplexeste und Logistik als redundanteste Elemente benannt.

Als überragende Schlüsselelemente der KRITIS in Krankenhäusern, welche zu einer Kettenreaktion führen können, wurden durch die Experten folgende benannt: Energieversorgung, Personal, Informationstechnologie.

### Entwicklung des finalen KRITIS-Modells

**Figure 2:**
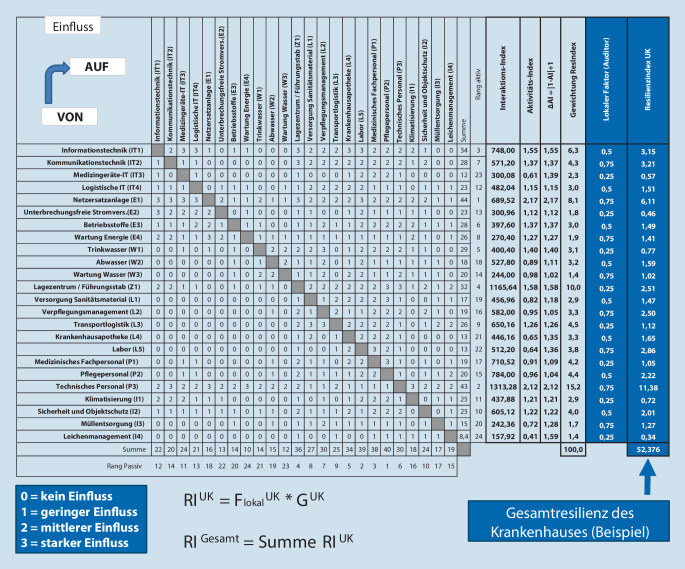

Aus den Mittelwerten der Expert*innenmeinungen konnten in der Einflussmatrix für alle Unterkategorien die Zeilen- und Spaltensummen sowie Gesamt-Scores ermittelt werden, die für die weiteren Berechnungen zur Modellbildung essenziell sind. Insgesamt sind in die Erstellung der finalen Matrix 6 Expertenaussagen zu je 552 Items, also 3312 Einzelwerte, eingeflossen. Aus den Zeilen- und Spaltensummen der Matrix lassen sich eine AS und eine PS für jede Unterkategorie der KRITIS ermitteln (Abb. 2). Am kritischsten sind jene Bereiche zu sehen, die auf beiden Skalen einen hohen Wert erreichen.

### Praktische Anwendung des Modells im Self-Assessment

Anhand der Bewertungen der befragten Expert*innen berechnet, ergibt sich für alle Unterkategorien der KRITIS im Krankenhaus eine Gewichtung (Abb. 2), die mithilfe eines selbst vergebenen lokalen Faktors zur Bestimmung des Resilienzindex eingesetzt werden kann.

Hierzu bewertet eine fachkundige Person vor Ort den tatsächlichen Zustand der jeweiligen Unterkategorie (nicht vorhanden: 0; deutliche Mängel: 0,25; moderate Mängel: 0,5; geringe Mängel: 0,75; keine Mängel: 1). Mit der Gewichtung multipliziert, ergibt dies den Indexwert für die Resilienz der Unterkategorie.

Der Resilienzindex der Kategorien *RI*^*K*^ ergibt sich aus Addition der jeweiligen der Kategorie zugeordneten Unterkategorien. Der Resilienzindex gesamt *RI*^*Ges*^ ist die Summe aller 7 Kategorien ${RI}_{1}^{K}$ bis ${RI}_{7}^{K}$ Dieser wird in einer gedrittelten 100-Punkte-Skala bewertet (gut/mittel/schlecht).

**Figure 3:**
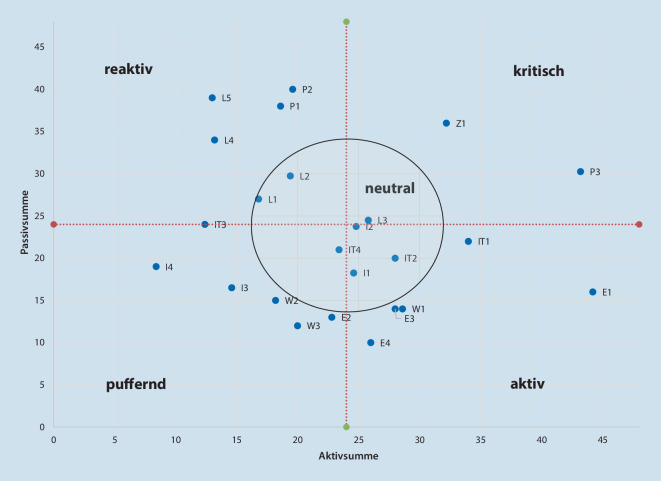

Zur Identifikation der Lageparameter wurde eine Visualisierung in Form eines Punktdiagramms nach Vester [20] gewählt (Abb. 3). Dies hilft dabei, durch Auswertung des rechten oberen Quadranten die Elemente mit besonderer Kritikalität auf einen Blick darstellen zu können.

Die Mittelwerte der dargestellten Aktiv- und Passivsummen liegen jeweils bei 24, daher wurden auch dort die Grenzen zwischen den Quadranten gezogen. Lagezentrum/Führungsstab (Z1) und Technisches Personal (P3) sind die kritischsten Unterkategorien, da sie sowohl auf andere Kategorien sehr hohen Einfluss haben als auch von anderen Kategorien beeinflusst werden. Die Versorgung mit Notstrom (E1) sowie Informationstechnik (IT1) wirken sich auf viele andere Unterkategorien aus, ohne selbst stark beeinflusst zu werden. Die Kategorien Personal (P) und Logistik (L) sind dominierend in der Passivsumme.

Die Ergebnisse zeigen, dass es für KRITIS im Krankenhaus mehrere Schlüsselelement gibt: Die Netzersatzanlage (E1) hat die größten Auswirkungen auf alle anderen Bereiche, und das Pflegepersonal (P2) ist für seine Arbeit am stärksten von allen anderen abhängig. Die kritischsten, weil am stärksten in das Gesamtsystem verflochtenen Elemente, sind Lagezentrum/Führungsstab (Z1) und Technisches Personal (P3), von denen die gesamte KRITIS im Krankenhaus abhängt.

Die Selbstbewertung hat für die Anwender*innen den Vorteil, dass im Gegensatz zur Auditierung nie dritte Personen Kenntnis über Vulnerabilitäten der Infrastruktur erlangen und auch keine Informationen nach außen gelangen. Diese Sicherheit versprechende Vorgehensweise erfordert allerdings geschultes Personal vor Ort und nimmt die Möglichkeit des fachlich-konstruktiven Austauschs. Der Gefahr einer Betriebsblindheit sollte somit durch einen Erfahrungsaustausch im vertraulichen Rahmen, z. B. innerhalb eines Klinikverbundes, begegnet werden.

## Diskussion

Die KRITIS kann nicht für alle potenziellen Szenarien vorgehalten werden, ohne hierbei irrational hohe Kosten zu generieren [16]. Ein Inputfaktor für Planungen müssen folglich immer auch die Rahmenbedingungen sein, in welchen ein Krankenhaus agiert. Das Szenario des absichtlich herbeigeführten Stromausfalls im Winter war exemplarisch sehr gut zur Bearbeitung der Forschungsfrage geeignet.

Für die Bewertung der KRITIS im komplexen System Krankenhaus ist neben der theoretischen Systemkenntnis auch viel Erfahrung unerlässlich. Das jeweilige Fachwissen zum Befüllen der Tabelle war breit gestreut und von einer hohen Subjektivität geprägt. Der deshalb gewählte semiquantitative Forschungsansatz zeigte erwartungsgemäß seine Grenzen in einer hohen Varianz bei der Interdependenzanalyse.

Die Untersuchung hat gezeigt, dass alle befragten Krankenhäuser ein sehr starkes Interesse an vernetzten Übungen haben, diese aber häufig an nicht verfügbaren Partnern scheitern.

Eine Akzeptanz der Planungsinstrumente in der Krankenhaus-Praxis ist nur dann gegeben, wenn Anwender unterschiedlichster Fachrichtungen und Qualifikationen mit wenig Aufwand verstehen können, wie Kennzahlen und Prozessmodelle zustande kommen. Der Schlüssel hierzu liegt in strikter Vereinfachung der Tools und stetiger Anpassung an sich verändernde Umstände.

Eine Einordnung in die Forschungslandschaft ist leider nicht möglich, da die vorliegende Studie Grundlagenforschung darstellt. Im Vergleich zum bereits weltweit etablierten HSI zeigten sich für das vorliegende Modell bei den Untersuchungsteilnehmern deutliche Anwendungsvorteile mit zunehmender Technisierung und Komplexität der Krankenhaus-Infrastruktur.

### Limitationen

Es gibt zahlreiche Variablen, die uns derzeit nicht bekannt sind. Ebenso sind nicht alle Faktoren vorhersagbar bzw. messbar. Ein Beispiel ist die Anzahl der Beschäftigten, die im Krisenfall nicht mehr am Arbeitsplatz erscheinen und damit das gesamte System gefährden. Hier kann man zwar Annahmen treffen, aber eine Befragung des Personals im Vorfeld hätte keinerlei Erfolg, da von einer sehr starken sozialen Erwünschtheit bei den Antworten auszugehen ist.

Die Durchführung einer vollständigen Sensitivitätsanalyse [17] verspricht bei der Bewertung der KRITIS keinen Erfolg, da die Zusammenhänge der einzelnen Kategorien nicht linear verlaufen, sondern dichotom sind. Zunehmende Digitalisierung und Verfügbarkeit von Daten aus technischen Geräten werden hier künftig mehr Potenzial für eine Analyse bieten, aber auch zu einer weiteren Verschiebung der Schwerpunkte in der KRITIS führen.

Trotz einer Quantifizierung von Aussagen handelt es sich noch immer um qualitative Daten, die der erstellten Matrix zugrunde liegen. Bei der Interpretation der Ergebnisse sind Verzerrungen (Bias) bei Teilnehmenden und Forschenden daher unbedingt zu berücksichtigen und ggf. durch quantitative Methoden zu validieren.

## Fazit für die Praxis

- Mit Blick auf das 2023 zu erwartende KRITIS-Dachgesetz erhalten Krankenhausbetreiber in Deutschland mit dem hier vorgestellten Modell ein Instrument des künftig verpflichtenden Risiko‑, Krisen und Resilienzmanagements.
- Der Resilienzindex kann dabei unterstützen, individuelle Schwachstellen in einem Krankenhaus zu erkennen und Alarmschwellen festzulegen, die selbstbestimmt aktives Handeln auslösen und Vertrauen fördern.
- Anhand der Übersicht der Kategorien und Unterkategorien können Katastrophenschutzverantwortliche im Krankenhaus reflektieren, ob in ihrer individuellen Notfallplanung alle Elemente der KRITIS berücksichtigt wurden, und darauf aufbauend eine Selbstbewertung vornehmen.
- Die erhaltenen Kennzahlen können ebenso ins QM eingebunden oder als Grundlage für die Finanzplanung des Bereiches Krisenvorsorge herangezogen werden.
- Die Forschungsergebnisse dienen der Entwicklung und Unterstützung einer Risikokompetenz bei den Entscheidern im Krankenhaus.
- Es empfiehlt sich eine Umsetzung der noch sehr komplexen Formeln und Tabellen in ein anwenderfreundliches Format wie beispielsweise eine App.

## Footnotes

1 Drei Versorgungsstufen plus Fachkliniken und drei Trägerschaften: öffentlich, frei-gemeinnützig, privat. Dies ergibt eine Auswahl von vier mal drei Kombinationen.

2 Die interviewende Person schreibt während der Interviews mit, lässt aber Inhalte mit Bezügen zu Namen, Daten und Orten aus Datenschutzgründen aus, indem diese umschrieben werden (z. B. „Firma X aus Y‑Stadt“).

3 Auswahl: 23 Datenbanken, z. B. MEDLINE, ScienceDirect, JSTOR Journals, Bibliotheksverbund Bayern, Academic Search Index etc.

4 Von 174 Suchergebnissen nach den genannten Kriterien wurden 154 nach Sichtung des Abstract und die übrigen 20 nach Sichtung des Volltextes ausgeschlossen.

## References

[1] Adams HA, Tecklenburg A (2007). Der Notfallplan des Krankenhauses: Grundlagen und allgemeine Struktur [The hospital emergency plan—basics and general structure]. Intensivmed Notfallmed.

[2] Bilban C (2019). Mythos „Gerasimov-Doktrin“: Ansichten des russischen Militärs oder Grundlage hybrider Kriegsführung?: eine Analyse der Rezeptionen in Europa und China.

[3] Bundesministerium des Innern (2009). Nationale Strategie zum Schutz kritischer Infrastrukturen (KRITIS-Strategie).

[4] Bundesministerium des Innern und für Heimat (2022). Eckpunkte für das KRITIS-Dachgesetz [Elektronische Ressource]: Ziele und Maßnahmen für die 20. Legislaturperiode.

[5] Dierich A, Bösche U, Wurbs S (2020) Analyse von Interdependenzen zwischen KRITIS: Empfehlungen für Praxisakteure aus Versorgungsunternehmen und kommunalen Behörden, 2. Aufl. Berlin

[6] Dungaciu D, Naumescu V (2015). The European Union’s Eastern neighbourhood today: Politics, dynamics, perspectives.

[7] Fekete A, Neisser F, Tzavella K, Hetkämper C (2019). Wege zu einem Mindestversorgungskonzept: Kritische Infrastrukturen und Resilienz.

[8] Flick U (2002). Qualitative Sozialforschung: Eine Einführung.

[9] Fuchs-Kittkowski K, Parthey H, Spur G (2007). Zur (informatischen) Modellbildung im Methodengefüge der Wissenschaft: Zur revolutionären Rolle der Methoden in der Wissenschaft. Wissenschaft und Technik in theoretischer Reflexion.

[10] Mayring P (2000) Qualitative Inhaltsanalyse. Forum Qualitative Sozialforsch 1(2). 10.17169/fqs-1.2.1089

[11] Petermann T, Bradke H, Lüllmann A, Poetzsch M, Riehm U (2011). Was bei einem Blackout geschieht: Folgen eines langandauernden und großflächigen Stromausfalls.

[12] Pfenninger E, Adolph O (2017). Memorandum – Zur Vulnerabilität kritischer Infrastrukturen an Bundesdeutschen Kliniken [Memorandum—On the vulnerability of critical infrastructures in German hospitals]. Notfall Rettungsmed.

[13] Pfenninger E, Güzelel H (2017). Folgen einer unzureichenden Krankenhaus-Katastrophenplanung: Betrachtung anhand eines Risikomodells [Impact assessment of inadequate hospital disaster management: Reflection based on a risk model. Anaesthesist.

[14] Probst GJB, Gomez P (1989). Vernetztes Denken: Unternehmen ganzheitlich führen.

[15] Ritz F (2015). Betriebliches Sicherheitsmanagement: Aufbau und Entwicklung widerstandsfähiger Arbeitssysteme.

[16] Romeike F (2018). Risikomanagement.

[17] Romeike F, Spitzner J (2013). Von Szenarioanalyse bis Wargaming: Betriebswirtschaftliche Simulationen im Praxiseinsatz.

[18] Scholtes K, Wurmb T, Rechenbach P (2018). Risiko- und Krisenmanagement im Krankenhaus: Alarm- und Einsatzplanung.

[19] Taleb NN (2013). Antifragilität: Anleitung für eine Welt, die wir nicht verstehen.

[20] Vester F (2019). Die Kunst vernetzt zu denken: Ideen und Werkzeuge für einen neuen Umgang mit Komplexität ; ein Bericht an den Club of Rome.

[21] Waitzinger SM (2015). A procedure model for risk identification in the development of technology driven business models.

